# Development of the Chinese preschooler dietary index: a tool to assess overall diet quality

**DOI:** 10.1186/s12889-022-14672-x

**Published:** 2022-12-26

**Authors:** Xiaoyu Wang, Yujie Xu, Bingbing Tan, Ruonan Duan, Shufang Shan, Linan Zeng, Kun Zou, Li Zhao, Jingyuan Xiong, Lingli Zhang, Shuang Rong, Guo Cheng

**Affiliations:** 1 Laboratory of Molecular Translational Medicine, Center for Translational Medicine, Key Laboratory of Birth Defects and Related Diseases of Women and Children (Sichuan University), Ministry of Education, Department of Pediatrics, West China Second University Hospital, Sichuan University, Chengdu, Sichuan 610041 People’s Republic of China; 2 Department of Nutrition and Food Safety, West China School of Public Health and West China Fourth Hospital, Sichuan University, Chengdu, China; 3 Shanxi Bethune Hospital, Shanxi Academy of Medical Sciences, Tongji Shanxi Hospital, Third Hospital of Shanxi Medical University, Taiyuan, China; 4 Tongji Hospital, Tongji Medical College, Huazhong University of Science and Technology, Wuhan, China; 5 Department of Pharmacy, Evidence-Based Pharmacy Center, Key Laboratory of Birth Defects and Related Diseases of Women and Children, Sichuan University), West China Second University Hospital, Sichuan University, Chengdu, China; 6 Department of Health Policy and Management, West China School of Public Health and West China Fourth Hospital, Sichuan University, Chengdu, China; 7 Healthy Food Evaluation Research Center, West China School of Public Health and West China Fourth Hospital,, Sichuan University, Chengdu, China; 8 Department of Nutrition Hygiene and Toxicology, Academic of Nutrition and Health, School of Public Health, Medical College, Wuhan University of Science and Technology, Wuhan, China

**Keywords:** Diet, Diet quality, Dietary index, Preschool children, China

## Abstract

**Background:**

Diet quality in early childhood has a long-term impact on health outcomes. However, there are scarce dietary indexes for Chinese preschool children, and the existing indexes had limited validity and reliability. This study thus aimed to develop a dietary index for preschool children based on the Chinese Dietary Guideline and Chinese Dietary Reference Intakes and to assess their overall diet quality using the China Health and Nutrition Survey (CHNS).

**Methods:**

The Chinese Preschooler Dietary Index (CPDI) included 11 components, covering 9 food group components and two nutrient components. The total scores of CPDI ranged from 0 to 90, with a higher score indicating greater diet quality. This study assessed the diet quality of 1742 preschoolers aged two to five years old from CHNS using the CPDI. Dietary intake data were obtained using three-day 24-h diet recalls, and sociodemographic information was also collected. Cochran-Mantel-Haensel (CMH) test was used to explore the association between demographic and CPDI total scores. The principal component analysis, correlation analysis and Cronbach’s alpha were used to evaluate the relative reliability and validity of the CPDI. Finally, a stepwise multiple regression analysis was performed to explore potential influencing factors of CPDI.

**Results:**

Among the 1742 CHNS preschool children, more than 70% resided in rural areas and 41.2% of the sample were raised in a low-income family. The mean CPDI score of the preschoolers was 38.8 ± 12.9. Higher diet scores were correlated with higher energy and nutrient intake. Children with higher age (β = 0.93, SE = 0.26, *P* = 0.0003), raised in a home with higher household income (β = 3.11, SE = 0.27, *P* < 0.0001) or living in urban areas (β = -4.44, SE = 0.66, *P* < 0.0001) were associated with higher CPDI scores.

**Conclusions:**

The CPDI is useful in evaluating the diet quality of preschool children. Based on the CPDI, the diet quality of Chinese preschoolers needs to be improved, especially in rural areas.

**Supplementary Information:**

The online version contains supplementary material available at 10.1186/s12889-022-14672-x.

## Background

Diet is essential to maintain good nutritional status and optimize health outcomes, especially in early childhood [1, 2]. Several studies have demonstrated the association of children’s dietary quality, evaluated by certain foods or nutrients, with growth and development [3–6]. However, a single or a few foods or nutrients could not capture the complexity of diet [7]. Dietary indexes which are developed based on national dietary guidelines or dietary recommendations, are widely used for accurate assessment of overall diet quality from 1980 to mid-2010 [8] A well designed dietary index could allow for monitoring dietary intake of children and quantifying the risk of chronic diseases caused by diet [9].

In western countries, several dietary indexes specifically for preschool children have been developed based on the main dietary problems of local preschoolers, by examining multiple dietary components [10] or adherence to dietary recommendations [11, 12], including the Finnish Children Healthy Eating Index in Finland [13], the Revised Children’s Diet Quality Index in America [10], the Preschoolers Diet-Lifestyle Index in Greece [11] and the Healthy Preference Index in Australia [12]. Due to the differences in dietary patterns and health issues of preschoolers in different countries, dietary indexes for western preschoolers may not be suitable for Chinese preschoolers. It is valuable to develop a dietary index specific to the needs of preschoolers in China.

To date, there are two dietary indexes for preschool children in China, i.e., the Chinese Healthy Eating Index (CHEI) [14] and the Chinese Diet Balance Index for preschoolers (DBI_C) [15]. According to the updated Chinese Dietary Guidelines (2016) [16], the CHEI was designed for Chinese aged two years and older, but its validity in evaluating diet quality of preschool children was poor [17], partly due to different dietary requirements and behavior varying among age groups. Although the DBI_C considered the scoring criteria for different age groups [15], its discrete and bidirectional scoring system limited the accuracy in capturing changes in foods or nutrients intake when the food intake is close to the cut-off values.

Therefore, to overcome existing limitations in current dietary indexes in China, the objective of this study was to establish a dietary index for Chinese preschool children (2–5 years old) based on the Chinese Dietary Guidelines (2016) [16] and Chinese Dietary Reference Intakes (DRIs) [18], and to assess the overall diet quality of Chinese preschool children from the China Health and Nutrition Survey (CHNS) [19].

## Methods

### Study sample

Data was obtained from the CHNS, an ongoing open cohort carried out by University of North Carolina and the Chinese Center for Disease Control and Prevention, which aimed to explore the health and nutritional status of Chinese population. The survey was conducted since 1989 with the data followed up and released every 2–4 years. About 7200 households with over 30,000 individuals in 12 provinces and municipal cities in China (Beijing, Chongqing, Guangxi, Guizhou, Heilongjiang, Henan, Hubei, Hunan, Jiangsu, Liaoning, Shandong, Shanghai) that vary substantially in geography, economic development, public resources, and health indicators were selected in the survey through a multistage, random cluster process [19]. The details of the CHNS have been published elsewhere [20].

In this study, 1808 preschool children (2–5 years old) with complete dietary data who participated in CHNS from 2004 to 2011 were included. Of these, 50 children with implausible energy intakes (< 400 kcal/d or > 4000 kcal/d) [21] and 16 children without complete potential covariates data (e.g. household size and per capita household income) were excluded. Therefore, this analysis was based on a final sample of 1742 preschoolers.

### Diet assessment and relevant variables

The dietary intake data was collected using 24-h dietary recall by trained investigators. Since there is large difference in energy intake of children between weekdays and weekends [22], three consecutive 24-h dietary recall were applied to cover both weekdays and weekends as possible. During the dietary assessment, parents or guardians were asked to recall children’s exact types and amounts of all the food items they consumed at home and away from home in the previous 24-h period. Standard plates and food models representing standard portion size for most of the food items, as well as validated food picture manual showing different amounts of food in standard scale were used to help participants better recall consumed amounts of foods and improve the accuracy of food intake estimates. Then the dietary intake data was converted into daily intake of energy and nutrients according to the Chinese Food Composition Tables (FCT) [23–25]. We calculated nutrient intakes by multiplying the nutrients amount of a standard serving of 100 g by the consumption of each food item, then average nutrient intakes from 3 day 24-h dietary recalls were generated. All data were independently calculated and entered the database by two research staff, and inconsistent results were verified by consensus.

The relevant variables in our study were age, sex, per capita household income, rural residence, and household size of participants, which were provided using questionnaires during the 24 h recalls by their parents. Per capita income in each survey year was inflated to values in 2015 by adjusting for consumer price index and then divided into five groups (yuan): low (≤ 5000), lower middle (5000–10,000), middle (10,000–20,000), upper middle (20,000–30,000) and high (> 30,000) [26].

### Chinese preschooler dietary index component selection

Selection of the Chinese Preschooler Dietary Index (CPDI) components was based on the key recommendations of the Chinese Dietary Guidelines (2016) [16] and the evidence for the association of diet with children’s health (Additional file 1: Table S1). The CPDI consisted of 11 components, including three aspects: five adequacy components, which have been recommended for sufficient consumption; five moderation components, referring to foods that should be consumed moderately; and one limitation components, whose intake should be reduced.

The adequacy components 1 to 5, vegetables, fruits, dairy and dairy products, soybeans and its products, aquatic products, and moderation components 1 to 3, cereals, eggs, red meat and poultry, were selected by the dietary intake recommendations of the Chinese Dietary Guidelines (2016) [16]. The age-specific reference value of each component was used to assess the consumption of food groups. Since the specific recommendations for whole grains, potatoes, and nuts for children aged 2–5 years old have not been proposed in China, they were not included as components in the CPDI.

The moderation components 4 and 5, vitamin A and iron, were selected based on recommendations on specific nutrients for preschoolers. Currently, vitamin A [27, 28] and iron deficiency [4, 29] remain the main nutritional issues of preschool children in China, especially in rural areas. It was essential to take the intake of vitamin A and iron into account in CPDI.

Consumption of high-sugar and high-fat snacks is common among preschool children in China [30]. And high-sugar and high-fat snacks has been suggested to increase the risk of dental caries [5], and cause excess energy then lead to overweight and obesity in children [31, 32]. Thus, high-sugar and high-fat snacks was included as the only limitation component in CPDI. High-sugar and high-fat snacks in CPDI were foods high in sugar and/or fat such as candy, chocolate, biscuits, cakes, potato chips, and popcorn.

### Component weighting and scoring of the Chinese preschooler dietary index

The maximum total score of CPDI was 90 points, in which a higher score indicated a better overall diet quality of preschool children. Considering that all kinds of foods contribute equally to diet, eight food group components were weighted for same scores in the CPDI, all of which were 0–10 points. Although high-sugar and high-fat snacks should be considered as same important with the main food groups, there were no evidence-based recommendation on its intake listed in the Chinese Dietary Guidelines (2016) [16]. Then the component of high-sugar and high-fat snacks was assigned five points. Given that food group components such as vegetables and meat could not reflect the intake of vitamin A and iron comprehensively, the CPDI incorporated vitamin A and iron as components and reduced their weights to avoid the collinearity between nutrients and other food group components..

The CPDI had a continuous scoring system. Children who did not meet the recommended intake level would be deducted corresponding points according to the degree of deviation. The scoring of CPDI was based on the food or nutrient density (amounts of foods or nutrients intake per 1000 cal). Since the daily recommended intake of various foods for preschool children were different between 2–3 years old (1000–1200 kcal/d) and 4–5 years old (1200–1400 kcal/d), the scoring of CPDI components was given specific scoring standards for the two age groups (Table 1).

**Table 1:** The scoring standard of Chinese Preschooler Dietary Index (CPDI)

Components	Range of score	2–3 years old		4–5 years old	
		**Criteria for maximum score**	**Criteria for minimum score**	**Criteria for maximum score**	**Criteria for minimum score**
**Adequacy components**^a^					
Vegetables	0–10	≥ 167 g/1000 kcal	0 g/1000 kcal	≥ 179 g/1000 kcal	0 g/1000 kcal
Fruits	0–10	≥ 83 g/1000 kcal	0 g/1000 kcal	≥ 107 g/1000 kcal	0 g/1000 kcal
Dairy and dairy products	0–10	≥ 417 g/1000 kcal	0 g/1000 kcal	≥ 250 g/1000 kcal	0 g/1000 kcal
Soybeans and its products	0–10	≥ 4 g/1000 kcal	0 g/1000 kcal	≥ 11 g/1000 kcal	0 g/1000 kcal
Aquatic products	0–10	≥ 12.5 g/1000 kcal	0 g/1000 kcal	≥ 14 g/1000 kcal	0 g/1000 kcal
**Moderation components**^b^					
Cereals	0–10	71 ~ 100 g/1000 kcal	0 or > 200 g/1000 kcal	71 ~ 125 g/1000 kcal	0 or > 250 g/1000 kcal
Eggs	0–10	17 ~ 25 g/1000 kcal	0 or > 50 g/1000 kcal	18 ~ 21 g/1000 kcal	0 or > 42 g/1000 kcal
Red meat and poultry	0–10	12.5 ~ 25 g/1000 kcal	0 or > 50 g/1000 kcal	18 ~ 33 g/1000 kcal	0 or > 66 g/1000 kcal
Vitamin A^c^	0–2.5	258 ~ 700 μg RAE^d^/1000 kcal	0 or > 1400 μg RAE^d^ /1000 kcal	257 ~ 750 μg RAE^d^ /1000 kcal	0 or > 1500 μg RAE^d^ /1000 kcal
Iron^c^	0–2.5	7.5 ~ 25 mg/1000 kcal	0 or > 50 mg/1000 kcal	7.1 ~ 25 mg/1000 kcal	0 or > 50 mg/1000 kcal
**Limitation components**^e^					
High-sugar and high-fat snacks^f^	0–5	0 g/1000 kcal	> 100 g/1000 kcal	0 g/1000 kcal	> 100 g/1000 kcal
**CPDI**^g^ **Total score**	0–90				

^a^Foods or nutrients that recommend consuming sufficiently

^b^Foods should be consumed moderately. Selecting consumption per 1000 kcal between the lowest and highest recommended amount as the standard of maximum score

^c^Based on the Chinese Dietary Reference Intakes 2013 [18] for vitamin A and iron

^d^RAE = Retinol Activity Equivalents

^e^Food intake should be reduced

^f^Snack foods high in sugar or fat (e.g. candy, chocolate, biscuits, cakes, potato chips, popcorn). The tolerable maximum intake of high-sugar and high-fat snacks was assigned based on the Optimized Mixed Diet (OMD) [33]

^g^CPDI = Chinese Preschooler Dietary Index

For adequacy components, scores were assigned the maximum score when the intake reached or exceeded the recommended intake level. If not, scores would be determined by the ratio of the actual intake and recommended intake as follows:

$$\frac{\mathbf{a}\mathbf{c}\mathbf{t}\mathbf{u}\mathbf{a}\mathbf{l}\,\mathbf{i}\mathbf{n}\mathbf{t}\mathbf{a}\mathbf{k}\mathbf{e}}{\mathbf{r}\mathbf{e}\mathbf{c}\mathbf{o}\mathbf{m}\mathbf{m}\mathbf{e}\mathbf{n}\mathbf{d}\mathbf{e}\mathbf{d}\,\mathbf{i}\mathbf{n}\mathbf{t}\mathbf{a}\mathbf{k}\mathbf{e}}\times \mathbf{m}\mathbf{a}\mathbf{x}\mathbf{i}\mathbf{m}\mathbf{u}\mathbf{m}\,\mathbf{s}\mathbf{c}\mathbf{o}\mathbf{r}\mathbf{e}$$

For example, children (5 years old) who consumed 200 g/1000 kcal dairy and dairy products received 8 points [ (200/250) × 10 = 8 points].

For moderation components, scores were assigned maximum score if the intake was within the recommended intake range. When the intake was 0 or more than twice the upper limit of recommended intake, the scores were 0. If the intake was higher than the upper limit or lower than the lower limit, scores were calculated as follows:

$$\left(1-\left|1-\frac{\mathbf{actual}\,\mathbf{intake}}{\mathbf{the}\,\mathbf{upper}\,\mathbf{limit}\,\mathbf{or}\,\mathbf{the}\,\mathbf{lower}\,\mathbf{limit}\,\mathbf{of}\,\mathbf{recommended}\,\mathbf{intake}}\right|\right)\times\mathbf m\mathbf a\mathbf x\mathbf i\mathbf m\mathbf u\mathbf m\,\mathbf s\mathbf c\mathbf o\mathbf r\mathbf e$$

For example, children (3 years old) who consumed 150 g/1000 kcal cereals received 5 points [ (1-|1–150/100|) × 10 = 5 points]. For vitamin A and iron, the recommended intake ranged from recommended nutrient intake (RNI) to tolerable upper intake level (UL) according to Chinese Dietary Reference Intakes (DRIs) [18], avoiding excessive intakes of nutrients.

For high-sugar and high-fat snacks, when the intake exceeded the limit intake, the score was 0 points. When the intake was less than the limit intake, the calculation formula was as follows:

$$\left(1-\frac{\mathbf{actual}\,\mathbf{intake}}{\mathbf{recommended}\,\mathbf{intake}}\right)\times5$$

Since there is no data on the limit intake of high-sugar and high-fat snacks, the recommended intake of “tolerated foods” (50 g/d) in the Optimized Mixed Diet [33] for German children aged 4–6 years was used as the intake standard for high-sugar and high-fat snacks in CPDI. For example, children (4 years old) who consumed 45 g/1000 kcal high-sugar and high-fat snacks received 2.75 points [ (1–45/100) × 5 = 2.75 points].

### Statistical analysis

All data processing and statistical analysis were performed using SAS 9.4. *P* values < 0.05 (two-tailed) were considered statistically significant. The total scores of CPDI were normally distributed, so the mean and standard deviation (SD) were used to present values. Mean dietary consumption and scores of CPDI components were described by medians (25th percentile, 75th percentile). To characterize the dietary quality of the sample, the continuous CPDI total scores were divided into low (< 25th percentile), medium (≥ 25th percentile and ≤ 75th percentile) and high (> 75th percentile). To assess the relative validity of the CPDI, principal component analysis (PCA) and Spearman correlation analysis was performed. Correlation analysis was used to test the association between CPDI total scores and nutrient adequacy ratios for energy intake and 8 nutrients not included in the CPDI and to test the correlations between component scores and the total score; while PCA was used to examine the dimensions of the CPDI. We also calculated the Cronbach coefficient alpha to test the internal consistency of CPDI. Moreover, the Cochran-Mantel-Haensel (CMH) test was used to compare the demographics feature (age, sex, residence and per capita household income) between different quartiles of CPDI scores of the preschoolers. Finally, to explore the influencing factors of CPDI, stepwise multiple linear regression analysis was used, and the variables selected in the regression that were thought to be related to diet quality in previous observations.

## Results

General characteristics of study participants in different diet quality levels are shown in Table 2. The mean age of these children was 3.6 ± 1.1 years, and 54.1% of them were boys. More than half of the participants lived in rural areas (72.0%) and had medium per capita household income (54.2%). The mean CPDI score was 38.8 ± 12.9. Children with older age, living in urban areas, and raised in high-income family had better dietary quality (*P* < 0.001). There was no statistical difference in the diet quality between male and female children in the present study (*P* = 0.4958).

**Table 2:** Characteristics of CHNS preschool children and the proportion of participants with different characteristics by categories of CPDI (%)^a^

**Characteristics**	**n (%)**	**CPDI Score**	**Diet Quality According to the CPDI, n (%)** ^b^			***p*-trend**^c^
			**Low**	**Medium**	**High**	
**Age**						
2 years -	393 (22.5)	37.7 ± 12.9	425 (24.4)	402 (23.1)	345 (19.8)	0.0076
3 years -	430 (24.7)	37.7 ± 12.9	472 (27.1)	434 (24.9)	380 (21.8)	
4 years -	435 (25.0)	39.7 ± 12.9	420 (24.1)	413 (23.7)	493 (28.3)	
5 years -	484 (27.8)	40.0 ± 12.8	425 (24.4)	493 (28.3)	524 (30.1)	
**Sex**						
Boy	955 (54.1)	38.9 ± 13.2	946 (54.3)	944 (54.2)	986 (56.6)	0.4958
Girl	787 (45.9)	38.7 ± 12.6	796 (45.7)	798 (45.8)	756 (43.4)	
**Residency**						
Urban	488 (28.0)	43.4 ± 13.5	345 (19.8)	406 (23.3)	798 (45.8)	< 0.0001
Rural	1254 (72.0)	37.1 ± 12.2	1397 (80.2)	1336 (76.7)	944 (54.2)	
**Per capita household income**^**d**^						
Low	718 (41.2)	34.9 ± 12.3	1030 (59.1)	711 (40.8)	420 (24.1)	< 0.0001
Lower middle	479 (27.5)	39.0 ± 12.1	413 (23.7)	521 (29.9)	460 (26.4)	
Middle	365 (21.0)	42.8 ± 12.8	235 (13.5)	341 (19.6)	542 (31.1)	
Upper middle	100 (5.7)	45.4 ± 11.3	28 (1.6)	115 (6.6)	145 (8.3)	
High	80 (4.6)	47.8 ± 12.5	36 (2.1)	54 (3.1)	175 (10.1)	

^a^*n* = 1742, *CHNS* China Health and Nutrition Survey, *CPDI* Chinese Preschooler Dietary Index

^b^Categories of the diet quality level according to the CPDI total scores: low (< 25th percentile), medium (≥ 25th percentile and ≤ 75th percentile), and high (> 75th percentile)

^c^*P* value for Cochran-Mantel-Haensel (CMH) test

^d^Per capita income in each survey year was inflated to values in 2015 by adjusting for consumer price index and then divided into five groups (yuan): low (≤ 5000), lower middle (5000–10,000), middle (10,000–20,000), upper middle (20,000–30,000) and high (> 30,000) [26]

We found that the correlations among component scores and total score for CPDI were quite low (0.02–0.35), indicating that single component could provide independent information (Table 3). The scree plot revealed that three factors with eigenvalue > 1 were present, accounting for 46.3% of the total variance in the index, which confirmed the multidimensional nature of CPDI and its capacity to reflect the complexity of diet quality (Additional file 1: Figure S1).

**Table 3:** Spearman’s correlation coefficient of the CPDI total score and component subscore

CPDI Component	Cereals	Vegetables	Fruits	Dairy and dairy products	Soy beans and its products	Aquatic products	Eggs	Red meat and poultry	High-sugar and high-fat snacks	Vitamin A	Iron	Total score^a^
Cereals	1.00											
Vegetables	-0.31*	1.00										
Fruits	0.07*	-0.02	1.00									
Dairy and dairy products	0.17*	-0.11*	0.36*	1.00								
Soybeans and its products	0.08*	0.06*	0.07*	0.07*	1.00							
Aquatic products	0.04	0.02	0.17*	0.18*	0.09*	1.00						
Eggs	0.04	-0.05*	0.05*	0.05*	0.07*	0.05*	1.00					
Red meat and poultry	0.13*	-0.17*	0.01	0.03	0.04	0.02	0.06*	1.00				
High-sugar and high-fat snacks	-0.14*	0.14*	-0.27*	-0.32*	-0.07*	-0.07*	-0.04	-0.08*	1.00			
Vitamin A	-0.06*	0.36*	0.26*	0.29*	0.08*	0.22*	-0.03	-0.06*	-0.16*	1.00		
Iron	-0.39*	0.46*	0.17*	0.12*	0.17*	0.17*	-0.03	-0.21*	-0.05*	0.45*	1.00	
Total score^a^	-0.05*	-0.05*	0.21*	0.25*	0.16*	0.20*	0.02	-0.03	-0.25*	0.33*	0.22*	1.00

^a^Total score minus the score of a specified component. * *P* < 0.05

The correlation of energy intake, fiber intake and nutrient adequacy ratio with the CPDI scores are presented in Table 4. The CPDI scores had positive correlations with daily energy intake (*r* = 0.23, *P* < 0.0001) and energy from protein (*r* = 0.33, *P* < 0.0001) and fat (*r* = 0.29, *P* < 0.0001). Fiber intake (*r* = 0.25, *P* < 0.0001), adequacy ratios of vitamins and minerals that not included in the CPDI (*r* = 0.22–0.50) were positively associated with the CPDI scores, indicating that increasing CPDI total scores reflected greater overall diet quality. Moreover, the CPDI had a standard and nonstandard Cronbach’s coefficient alpha of 0.53 and 0.60, respectively.

**Table 4:** Correlation between nutrient intakes and diet quality assessed by the Chinese Preschooler Dietary Index (CPDI) in the present study^a^

	**Diet Quality According to CPDI**^b^			**r**^c^	***P* value**^d^
	**Low**	**Medium**	**High**		
n	435	872	435		
Energy (kcal/day)	927.2 (706.0, 1217.6)	998.2 (763.3, 1276.0)	1151.7 (917.2, 1459.1)	0.23	< 0.0001
Protein (% energy)	10.7 (8.7, 12.8)	12.2 (10.0, 14.3)	13.9 (11.5, 17.0)	0.33	< 0.0001
Fat (% energy)	12.3 (4.3, 22.8)	17.8 (11.4, 24.4)	21.0 (15.7, 27.9)	0.29	< 0.0001
Carbohydrates (% energy)	55.5 (44.0, 66.8)	50.5 (41.3, 60.5)	47.0 (38.8, 55.2)	-0.20	< 0.0001
Fiber (g/day)	3.2 (2.1, 4.9)	3.6 (2.4, 5.2)	4.7 (3.2, 6.5)	0.25	< 0.0001
**Nutrient adequacy ratio** ^e^					
Thiamine	0.5 (0.4, 0.8)	0.6 (0.4, 0.8)	0.7 (0.5, 1.0)	0.22	< 0.0001
Riboflavin	0.4 (0.3, 0.6)	0.6 (0.4, 0.8)	0.9 (0.6, 1.0)	0.44	< 0.0001
Niacin	0.7 (0.5, 1.0)	0.8 (0.6, 1.0)	1.0 (0.7, 1.0)	0.25	< 0.0001
Vitamin C	0.4 (0.2, 0.8)	0.6 (0.3, 1.0)	0.9 (0.6, 1.0)	0.35	< 0.0001
Vitamin E	0.5 (0.3, 0.7)	0.6 (0.4, 0.9)	1.0 (0.7, 1.0)	0.47	< 0.0001
Calcium	0.2 (0.1, 0.2)	0.2 (0.2, 0.4)	0.4 (0.3, 0.7)	0.50	< 0.0001
Potassium	0.5 (0.3, 0.7)	0.6 (0.5, 0.9)	0.9 (0.7, 1.0)	0.46	< 0.0001
Zinc	0.8 (0.5, 1.0)	0.9 (0.7, 1.0)	1.0 (0.9, 1.0)	0.31	< 0.0001

^a^*n* = 1742; values were presented in the form of median (25th percentile, 75th percentile)

^b^Categories of the diet quality level according to the CPDI total scores: low (< 25th percentile), medium (≥ 25th percentile and ≤ 75th percentile), and high (> 75th percentile)

^c^Spearman correlation coefficients between CPDI and other parameters

^d^*P* value for the Spearman correlation

^e^Nutrient adequacy ratio = daily actual intakes /recommended amount of nutrient according to the Chinese Dietary Reference Intakes [18], and each truncated at 1

The mean consumption and sub-score of each component of the CPDI are presented in Additional file 1: Table S2. Among the CPDI components, the highest mean sub-scores were observed for iron, high-sugar and high-fat snacks, and soybeans and its products (iron for 2.5, high-sugar and high-fat snacks for 5.0, soybeans and its products for 8.1), indicating the consumption of these foods/nutrients met the requirements to a certain extent. There were insufficient intakes of fruits, dairy and aquatic products and excessive intakes of red meat and poultry in Chinese preschool children (with sub-score 0 points). More than 80% of the participants consumed vegetables below the recommended intake level and only 3% of participants met the recommended intake of vitamin A, with the mean sub-scores for vegetables and vitamin A being 5.9 and 0.9, respectively.

Multiple stepwise regression analysis was used to explore the factors (Per capita household income, residence, age, sex, and household size) affecting the CPDI (Table 5). The final model indicated that per capita household income, residence and age were significantly associated with the CPDI. The rural residence was the strongest factor associated with lower CPDI scores of children (*β* = -4.44, SE = 0.66, *P* < 0.0001). Additionally, Children with higher per capita household income (*β* = 3.11, SE = 0.27, *P* < 0.0001) or older age (*β* = 0.93, SE = 0.26, *P* = 0.0003) received higher CPDI scores.

**Table 5:** Stepwise multiple regression analysis of factors affecting the Chinese Preschooler Dietary Index (CPDI)^a^

Variables	Stepwise Multiple Model		
	***β***	**SE**^**b**^	***P* value**^**c**^
Constant	36.75	1.69	< 0.0001
Per capita household income^d^	3.11	0.27	< 0.0001
Residence^e^	-4.44	0.66	< 0.0001
Age	0.93	0.26	0.0003

^a^Excluded variables: sex and household size

^b^*SE* Standard Error

^c^*P* value for stepwise multiple model

^d^Per capita income in each survey year was inflated to values in 2015 by adjusting for consumer price index and then divided into five groups (yuan): low (≤ 5000) (reference group), lower middle (5000–10,000), middle (10,000–20,000), upper middle (20,000–30,000) and high (> 30,000)[26]

^e^Residence included urban and rural areas, urban as the reference group

## Discussion

In this study, we aimed to establish a Chinese Preschooler Dietary Index to evaluate the diet quality of Chinese preschool children. Our results supported the reliability and validity of CPDI as a valuable index to measure diet quality of preschoolers aged 2 to 5 years. Dietary quality of the CHNS preschoolers needs to be improved as the average CPDI score (38.8 ± 12.9) was less than half of the full score. In addition, residence, age and per capita household income were the potential influencing factors for CPDI.

Based on the Chinese Dietary Guidelines (2016) and Chinese Dietary Reference Intakes (DRIs), the key recommendations of food groups were included in the CPDI to evaluate their association with daily consumption of preschoolers. Since iron or vitamin A deficiency are common among Chinese preschoolers [28, 34], they were incorporated in the CPDI. Given that dietary diversity can be reflected by multiple food components to a certain extent and collinearity may exist between the food variety and other food components [35], food variety or dietary diversity was not selected as a component in the CPDI. However, as other dietary indexes established earlier showed [36, 37], the importance of dietary diversity for healthy eating still could not be ignored. Compared with the discrete scoring system in DBI_C, CPDI adopted a continuous scoring system to improve accuracy in capturing changes in foods or nutrients intake when the food intake is close to the cut-off values [35], which more effectively reflects the adherence to dietary recommendations. Furthermore, the CPDI was capable to reflect the complexity of diet patterns due to the fact that no single ingredient drove the total score, as well as significantly correlated with the intakes of foods and nutrients included in the index, and with intakes of other essential nutrients These associations were consistent with dietary quality indexes developed for children in Finland [13], America [10] and Greece [11], which indicated that the CPDI could be used as a valuable tool to evaluate overall dietary quality of preschool children in China. As for the reliability of CPDI, the Cronbach alpha 0.53 and 0.60 was acceptable compared with other dietary indexes developed in other countries with alpha ranging from 0.22 to 0.68 [36, 38–41] Overall, the CPDI had relatively good reliability and validity.

The CPDI was used to assess overall dietary quality of the study participants from CHNS. Results from these CHNS children aged 2–5 years indicated that their dietary quality needs to be improved as the average CPDI score of the sample was less than half of the full score, which was partly consistent with dietary imbalances among Chinese preschoolers reported in the previous study (38.8 point of 0 to 90 point vs -17 point of -60 to 36 point) [15]. Among the 11 components in the CPDI, it is remarkable that Chinese preschoolers got a perfect score for high-sugar and high-fat snacks. Our results was similar to the last reported snacks consumption among Chinese preschoolers [42] from 12 provinces, partly due to the sample distribution in residence. There were 60% of children in the mentioned study and 72% of children in our study from rural areas, in which children were less accessible for snacks. Since diet data were collected from parents, who tended to report a lower level of snack consumption, social bias could not be ignored. Moreover, due to limited recommendation in snacks intake, we have only assigned five points for this item in the CPDI, caution should be paid when applying CPDI to measure the dietary quality of snacks. In addition, it is noteworthy that preschoolers had inadequate intakes of fruits, dairy, aquatic products and eggs, as well as excessive intakes of red meat and poultry, which was consistent with published studies in China [43, 44]. We found that 69.3% of participants did not consume dairy for three consecutive days, and the average intake of dairy was 53.0 ± 105.6 g/d. Evidence reported that dairy and its products could reduce the risk of overweight and obesity [45] and increase bone mineral content [46] in children. Therefore, preschool children should continue to increase the consumption of dairy and its products, especially those in rural areas. The dietary iron intake of Chinese preschoolers was relatively ideal in the present study, which was similar to the result of preschool children using RC-DQI [10]. The increased intake of dietary iron may be associated with the high consumption of red meat in recent years, which is conducive to improve the iron deficiency status of Chinese children [47].

Sociodemographic factors associated with the CPDI scores of children were also identified in this study. Similar to the findings among preschool children in Greece [48], older preschool children had better diet quality, and reasons may be as follows. Preschool age is a critical nutritional period that the dietary patterns change from breast milk and complementary foods to a variety of solid foods [49], younger preschoolers may have obstacles in accepting new foods. Unhealthy eating behaviors such as picky eating refers to that a child regularly had food preferences and refused to eat certain foods may be encountered in this period. When they grow older, most preschool children would enter childcare centers, in which educational activities and environmental intervention based on food policy will promote their eating behaviors [50] and dietary quality [51]. Meanwhile, we found that preschool children who were from a household with higher income had better diet quality scores, which was consistent with other research findings [52, 53]. This may be based on the fact that high-income families tend to pay more attention to food choices and have easier access to healthy foods [54].

The CPDI has several strengths. First, since the differences in individual energy intake were not considered in the previous dietary index [15], excessive consumption of foods may lead to excess energy intake. Therefore, the CPDI assessed the food groups and nutrients consumption by food or nutrient density [55], making the evaluation of individual dietary status depending on quality rather than quantity. Second, the CPDI lies in the fact that it was developed based on the recommended dietary intake and energy requirements of children in different age groups, which is beneficial to address dietary issues specific to subgroups of preschoolers. Third, we used the CPDI to examine the dietary quality of Chinese preschool children from the CHNS (2004–2011) covering both urban and rural areas in 12 provinces and municipal cities of China.

Several limitations need to be noted in this study. First, there may be information bias because the dietary data of preschool children was collected through the three days 24-h diet recalls, and long-term dietary intake cannot be reflected. Meanwhile, there are different combination of the three-day diet collection (43.1% of the sample had three weekdays, 39.4% had two weekdays and one weekend day, and 17.5% had one weekday and two weekend days), which might lead to bias in individual dietary intake. Seasonal differences in food choice and intake may also exist. Second, there may be some social bias in the self-reported data, for example, parents tended to report a lower level of high-sugar and high-fat snacks. Third, due to unavailable recommended intake range for high-sugar and high-fat snacks in Chinese preschoolers, we have assigned five points to the component, which might lead to underrated on its importance on health outcomes. Since the CHNS was not designed to be representative of China but to capture a range of economic and demographic circumstances from South to North, the generalizability of these participants was limited. However, the provinces and municipal cities in our sample have covered a large part of urban and rural areas in China. Finally, it would be more accurate to use biomarkers to assess the intake of iron and vitamin A, but the collection of blood and urine samples from preschool children is difficult in most large observational studies.

## Conclusions

The Chinese Preschooler Dietary Index was successfully established as an instrument to measure the overall diet quality and distinguish the differences in diet quality among Chinese preschool children. The diet quality of Chinese preschoolers is suboptimal and warrants improvement, especially for children in rural areas and low-income families. The CPDI can determine overconsumption as well as insufficient intake, which is beneficial for future research examining the associations between diet and health outcomes in preschool children. In addition, more validation and replicability are needed to advance this research in China.

## Supplementary Information

**Additional file 1: Table S1.** Components and weighting of Chinese Preschooler Dietary Index and the corresponding research evidence. **Table S2.** Consumption of the Chinese Preschooler Dietary Index (CPDI) components and the sub-scores for the quartiles of total CPDI score in the present study^a^. **Figure S1.** Scree plot from the principal component analysis of the CPDI.

## Data Availability

The datasets used and/or analyzed during the current study are available from the corresponding author on reasonable request.

## Abbreviations

CHNS: China Health and Nutrition Survey
CPDI: Chinese Preschooler Dietary Index
CHEI: Chinese Healthy Eating Index
CMH: Cochran-Mantel-Haensel
DBI_C: Chinese Diet Balance Index for preschool children
FCHEI: Finnish Children Healthy Eating Index
RC-DQI: Children’s Diet Quality Index
