# Supplementary material for: Development of the Chinese preschooler dietary index: a tool to assess overall diet quality
Source: BMC Public Health. 2022 Dec 26;22:2428. doi: 10.1186/s12889-022-14672-x (PMC9791773; doi:10.1186/s12889-022-14672-x)
Supplement: Supplementary file 1 — Additional file 1: Table S1. Components and weighting of Chinese Preschooler Dietary Index and the corresponding research evidence. Table S2. Consumption of the Chinese Preschooler Dietary Index (CPDI) components and the sub-scores for the quartiles of total CPDI score in the present studya. Figure S1. Scree plot from the principal component analysis of the CPDI. [file 12889_2022_14672_MOESM1_ESM.docx]

**Supplementary files**

**Additional file 1: Table S1**. Components and weighting of Chinese Preschooler Dietary Index and the corresponding research evidence.

| **Components** | **Weighting** | **Nutritional value** | **Research Evidence** |
| --- | --- | --- | --- |
| Cereals | 10 | Cereals are the most economical energy source for human, which are rich in carbohydrates and B vitamins. | Refined grains are positively associated with childhood obesity(1) and early puberty(2). |
| Vegetables | 10 | Vegetables are low in energy but high in vitamins, dietary fiber and plant compounds. | Vegetables are of great significance to prevent children’s obesity(3, 4)and hypertension(5), and to increase bone mass of children(6). |
| Fruits | 10 | Fruits are important sources of vitamin C, potassium, magnesium and dietary fiber. The organic acids in fruits are conducive to food digestion. | Sufficient intakes of fruits can reduce the risk of childhood obesity(7-9) and hypertension(5). |
| Dairy and dairy products | 10 | Dairy is rich in dietary calcium, high-quality protein and B vitamins. | Dairy can reduce the risk of obesity(10) and increase the bone mineral content in children(11). It can promote the growth and development of children(12, 13). |
| Soybeans and its products | 10 | Soybeans and its products are rich in high-quality plant protein, essential fatty acids, soy isoflavones and vitamin E. | Soybeans are important to prevent early puberty(14, 15) and breast cancer in adulthood(16). |
| Aquatic products | 10 | In addition to high-quality protein, minerals and vitamins, aquatic products also contain a lot of n-3 polyunsaturated fatty acids. | Aquatic products can promote children’s cognitive functions(17, 18) and retina development(19). However, the insufficient intakes of aquatic products is common in China(20). |
| Eggs | 10 | Eggs are the optimal sources of protein and rich in various vitamins. | Excessive egg intakes may be related to childhood obesity(21, 22). |
| Red meat and poultry | 10 | Red meat and poultry are rich in high-quality protein, fat-soluble vitamins, B vitamins and minerals. | There are positive associations of red meat and poultry with childhood central obesity(23) and early puberty(24) |
| High-sugar and high-fat snacks^a^ | 5 | High-sugar and high-fat snacks are high-energy and low-nutrient foods that contain a lot of added sugar and saturated fat. Added sugars are pure energy foods that have a high glycemic index, and excessive fat intake is likely to cause excess energy. | Excessive intake of high-sugar and high-fat snacks can lead to dental caries(25), overweight and obesity(26, 27) and cardiovascular disease(28) in children. Consumption of unhealthy snacks may increase the risk of mental distress and violent behaviors in children(29). |
| Vitamin A | 2.5 | Vitamin A can regulate cell growth and differentiation, and it plays an important role in the growth and development of children. | Vitamin A can reduce the risk of immunodeficiency and anemia. However, the intake of vitamin A is insufficient among Chinese preschool children(30-32). |
| Iron | 2.5 | Iron deficiency not only affects the body’s metabolic process but also reduces hemoglobin synthesis, inducing iron deficiency anemia. | At present, iron deficiency anemia is a major nutritional problem for preschool children in China, especially in rural areas(33, 34). |

^a^ Snack foods are high in sugar or fat (e.g. candy, chocolate, biscuits, cakes, potato chips, popcorn).

**Additional file 1: Table S2**. Consumption of the Chinese Preschooler Dietary Index (CPDI) components and the sub-scores for the quartiles of total CPDI score in the present study ^a^

| **Components** | **Amount consumed** | **Requirement**  **fulfilment (n/%)** | **CPDI subscore** | **CPDI Total Score** | | | |
| --- | --- | --- | --- | --- | --- | --- | --- |
|  |  |  |  | **Quartile 1** | **Quartile 2** | **Quartile 3** | **Quartile 4** |
| Vegetables (g/day) | 106.7 (61.7, 166.7) | 212 (12.2) | 5.9 (3.5, 9.1) | 5.8 (2.8, 9.5) | 6.0 (3.6, 9.0) | 5.3 (3.5, 8.5) | 6.4 (4.2, 9.2) |
| Fruits (g/day) | 0.0 (0.0, 76.7) | 282 (16.2) | 0.0 (0.0, 7.4) | 0.0 (0.0, 0.0) | 0.0 (0.0, 4.9) | 2.8 (0.0, 8.7) | 7.1 (2.7, 10.0) |
| Dairy and dairy products (g/day) | 0.0 (0.0, 66.7) | 25 (1.4) | 0.0 (0.0, 1.8) | 0.0 (0.0, 0.0) | 0.0 (0.0, 0.0) | 0.0 (0.0, 2.0) | 2.0 (0.0, 5.4) |
| Soybeans and its products (g/day) | 6.7 (0.0, 33.3) | 158 (9.1) | 8.1 (0.0, 10.0) | 0.0 (0.0, 0.0) | 4.3 (0.0, 10.0) | 10.0 (0.0, 10.0) | 10.0 (9.9, 10.0) |
| Aquatic products, g/day | 0.0 (0.0, 16.7) | 431 (24.7) | 0.0 (0.0, 10.0) | 0.0 (0.0, 0.0) | 0.0 (0.0, 0.0) | 0.0 (0.0, 10.0) | 10.0 (0.0, 10.0) |
| Cereals (g/day) | 150.0 (108.3, 210.0) | 62 (3.6) | 6.3 (2.2, 9.3) | 2.4 (0.0, 6.6) | 5.5 (2.1, 8.2) | 6.8 (4.0, 9.7) | 8.6 (6.2, 10.0) |
| Eggs (g/day) | 16.7 (0.0, 40.0) | 74 (4.3) | 0.0 (0.0, 6.8) | 0.0 (0.0, 0.0) | 0.0 (0.0, 5.5) | 3.2 (0.0, 7.4) | 5.5 (0.0, 9.0) |
| Red meat and poultry (g/day) | 40.0 (15.0, 75.0) | 106 (6.1) | 0.0 (0.0, 7.1) | 0.0 (0.0, 0.0) | 0.0 (0.0, 6.0) | 3.0 (0.0, 8.7) | 5.2 (0.0, 9.4) |
| Vitamin A (μg RAE/day) | 104.9 (52.8, 180.9) | 101 (5.8) | 0.9 (0.5, 1.5) | 0.6 (0.3, 1.3) | 0.8 (0.4, 1.4) | 1.0 (0.6, 1.6) | 1.3 (0.8, 1.9) |
| Iron (mg/day) | 7.7 (5.6, 10.3) | 522 (30.0) | 2.5 (2.1, 2.5) | 2.5 (2.0, 2.5) | 2.5 (1.9, 2.5) | 2.5 (2.1, 2.5) | 2.5 (2.3, 2.5) |
| High-sugar and high-fat snacks^b^ (g/day) | 0.0 (0.0, 16.7) | 1561 (89.7) | 5.0 (4.3, 5.0) | 5.0 (5.0, 5.0) | 5.0 (5.0, 5.0) | 5.0 (4.2, 5.0) | 5.0 (3.7, 5.0) |

^a^ n=1742; values were presented in the form of median (25th percentile, 75th percentile).

^b^ Snack foods high in sugar or fat (e.g. candy, chocolate, biscuits, cakes, potato chips, popcorn).

**Additional file 1: Figure S1. Scree plot from the principal component analysis of the CPDI.**


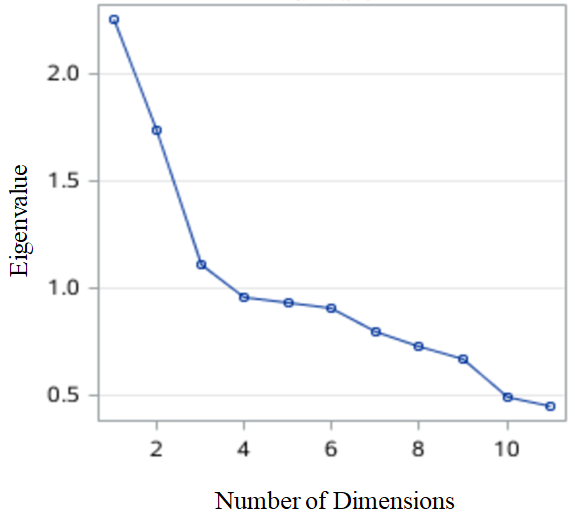
Factor-loading matrix for the three factors with eigenvalue >1 underline the CPDI

|  | Factor 1 | Factor 2 | Factor 3 |
| --- | --- | --- | --- |
| Cereals | -0.62 | 0.28 | 0.15 |
| Vegetables | 0.78 | -0.08 | 0.04 |
| Fruits | 0.05 | 0.68 | 0.02 |
| Dairy and dairy products | -0.03 | 0.72 | 0.04 |
| Soybeans and its products | 0.11 | 0.09 | 0.66 |
| Aquatic products | 0.14 | 0.34 | 0.39 |
| Eggs | -0.07 | -0.12 | 0.62 |
| Red meat and poultry | -0.35 | -0.02 | 0.39 |
| High-sugar and high-fat snacks | 0.10 | -0.56 | 0.04 |
| Vitamin A | 0.54 | 0.52 | 0.05 |
| Iron | 0.78 | 0.24 | 0.14 |

**References**

1. Dos Santos Rocha A, de Cássia Ribeiro-Silva R, Nunes de Oliveira Costa G, Alexandrina Figueiredo C, Cunha Rodrigues L, Maria Alvim Matos S, Leovigildo Fiaccone R, Oliveira PR, Alves-Santos NH, et al. Food Consumption as a Modifier of the Association between Gene Variants and Excess Body Weight in Children and Adolescents: A Study of the SCAALA Cohort. Nutrients. 2018;10.

2. Jansen EC, Zhou L, Perng W, Song PX, Rojo MMT, Mercado A, Peterson KE, Cantoral A. Vegetables and lean proteins-based and processed meats and refined grains -based dietary patterns in early childhood are associated with pubertal timing in a sex-specific manner: a prospective study of children from Mexico City. Nutrition research (New York, NY). 2018;56:41-50.

3. Kepper M, Tseng TS, Volaufova J, Scribner R, Nuss H, Sothern M. Pre-school obesity is inversely associated with vegetable intake, grocery stores and outdoor play. Pediatric obesity. 2016;11:e6-e8.

4. Vernarelli JA, Mitchell DC, Hartman TJ, Rolls BJ. Dietary energy density is associated with body weight status and vegetable intake in U.S. children. The Journal of nutrition. 2011;141:2204-10.

5. Shi L, Krupp D, Remer T. Salt, fruit and vegetable consumption and blood pressure development: a longitudinal investigation in healthy children. The British journal of nutrition. 2014;111:662-71.

6. Wosje KS, Khoury PR, Claytor RP, Copeland KA, Hornung RW, Daniels SR, Kalkwarf HJ. Dietary patterns associated with fat and bone mass in young children. The American journal of clinical nutrition. 2010;92:294-303.

7. Fletcher S, Wright C, Jones A, Parkinson K, Adamson A. Tracking of toddler fruit and vegetable preferences to intake and adiposity later in childhood. Maternal & child nutrition. 2017;13.

8. Miller P, Moore RH, Kral TVE. Children's daily fruit and vegetable intake: associations with maternal intake and child weight status. Journal of nutrition education and behavior. 2011;43:396-400.

9. Mandal B, Powell LM. Child care choices, food intake, and children's obesity status in the United States. Economics and human biology. 2014;14:50-61.

10. Dror DK. Dairy consumption and pre-school, school-age and adolescent obesity in developed countries: a systematic review and meta-analysis. Obesity reviews : an official journal of the International Association for the Study of Obesity. 2014;15:516-27.

11. de Lamas C, de Castro MJ, Gil-Campos M, Gil Á, Couce ML, Leis R. Effects of Dairy Product Consumption on Height and Bone Mineral Content in Children: A Systematic Review of Controlled Trials. Advances in nutrition (Bethesda, Md). 2019;10:S88-S96.

12. Duan Y, Pang X, Yang Z, Wang J, Jiang S, Bi Y, Wang S, Zhang H, Lai J. Association between Dairy Intake and Linear Growth in Chinese Pre-School Children. Nutrients. 2020;12.

13. Herber C, Bogler L, Subramanian SV, Vollmer S. Association between milk consumption and child growth for children aged 6-59 months. Scientific reports. 2020;10:6730.

14. Cheng G, Buyken AE, Shi L, Karaolis-Danckert N, Kroke A, Wudy SA, Degen GH, Remer T. Beyond overweight: nutrition as an important lifestyle factor influencing timing of puberty. Nutrition reviews. 2012;70:133-52.

15. Cheng G, Remer T, Prinz-Langenohl R, Blaszkewicz M, Degen GH, Buyken AE. Relation of isoflavones and fiber intake in childhood to the timing of puberty. The American journal of clinical nutrition. 2010;92:556-64.

16. Messina M, Rogero MM, Fisberg M, Waitzberg D. Health impact of childhood and adolescent soy consumption. Nutrition reviews. 2017;75:500-15.

17. Lehner A, Staub K, Aldakak L, Eppenberger P, Rühli F, Martin RD, Bender N. Fish consumption is associated with school performance in children in a non-linear way: Results from the German cohort study KiGGS. Evolution, medicine, and public health. 2020;2020.

18. Teisen MN, Vuholm S, Niclasen J, Aristizabal-Henao JJ, Stark KD, Geertsen SS, Damsgaard CT, Lauritzen L. Effects of oily fish intake on cognitive and socioemotional function in healthy 8-9-year-old children: the FiSK Junior randomized trial. The American journal of clinical nutrition. 2020;112:74-83.

19. Rogers LK, Valentine CJ, Keim SA. DHA supplementation: current implications in pregnancy and childhood. Pharmacological research. 2013;70:13-9.

20. Huang L, Wang Z, Wang H, Zhao L, Jiang H, Zhang B, Ding G. Nutrition transition and related health challenges over decades in China. European journal of clinical nutrition. 2021;75:247-52.

21. Yang YX, Wang XL, Leong PM, Zhang HM, Yang XG, Kong LZ, Zhai FY, Cheng YY, Guo JS, Su YX. New Chinese dietary guidelines: healthy eating patterns and food-based dietary recommendations. Asia Pac J Clin Nutr. 2018;27:908-13.

22. Song Y, Zhang X, Ma J, Zhang B, Hu P-j, Dong B. [Behavioral risk factors for overweight and obesity among Chinese primary and middle school students in 2010]. Zhonghua yu fang yi xue za zhi [Chinese journal of preventive medicine]. 2012;46:789-95.

23. Bradlee ML, Singer MR, Qureshi MM, Moore LL. Food group intake and central obesity among children and adolescents in the Third National Health and Nutrition Examination Survey (NHANES III). Public health nutrition. 2010;13:797-805.

24. Jansen EC, Marín C, Mora-Plazas M, Villamor E. Higher Childhood Red Meat Intake Frequency Is Associated with Earlier Age at Menarche. The Journal of nutrition. 2015;146:792-8.

25. Al-Dlaigan YH, Al-Meedania LA, Anil S. The influence of frequently consumed beverages and snacks on dental erosion among preschool children in Saudi Arabia. Nutrition journal. 2017;16:80.

26. Poorolajal J, Sahraei F, Mohamdadi Y, Doosti-Irani A, Moradi L. Behavioral factors influencing childhood obesity: a systematic review and meta-analysis. Obesity research & clinical practice. 2020;14:109-18.

27. Welsh JA, Cunningham SA. The role of added sugars in pediatric obesity. Pediatric clinics of North America. 2011;58.

28. Bel-Serrat S, Mouratidou T, Börnhorst C, Peplies J, De Henauw S, Marild S, Molnár D, Siani A, Tornaritis M, et al. Food consumption and cardiovascular risk factors in European children: the IDEFICS study. Pediatric obesity. 2013;8:225-36.

29. Zahedi H, Kelishadi R, Heshmat R, Motlagh ME, Ranjbar SH, Ardalan G, Payab M, Chinian M, Asayesh H, et al. Association between junk food consumption and mental health in a national sample of Iranian children and adolescents: the CASPIAN-IV study. Nutrition (Burbank, Los Angeles County, Calif). 2014;30:1391-7.

30. Zhang Y, Du Z, Ma W, Chang K, Zheng C. Vitamin A status and recurrent respiratory infection among Chinese children: A nationally representative survey. Asia Pacific journal of clinical nutrition. 2020;29:566-76.

31. Song P, Wang J, Wei W, Chang X, Wang M, An L. The Prevalence of Vitamin A Deficiency in Chinese Children: A Systematic Review and Bayesian Meta-Analysis. Nutrients. 2017;9.

32. Zhang Z-H, Ni M, Hu Y. [Current status of vitamin A deficiency in preschool children in Dongguan, China and the effect of vitamin A on serum ferritin and red blood cell parameters]. Zhongguo dang dai er ke za zhi = Chinese journal of contemporary pediatrics. 2018;20:195-9.

33. Chen K, Zhang X, Li T-Y, Chen L, Qu P, Liu Y-X. Co-assessment of iron, vitamin A and growth status to investigate anemia in preschool children in suburb Chongqing, China. World journal of pediatrics : WJP. 2009;5:275-81.

34. Wong AYS, Chan EW, Chui CSL, Sutcliffe AG, Wong ICK. The phenomenon of micronutrient deficiency among children in China: a systematic review of the literature. Public health nutrition. 2014;17:2605-18.
